# Of the importance of the clinical phenotypes in the interpretation of the studies dealing with Fabry disease

**DOI:** 10.1186/s13023-018-0979-z

**Published:** 2019-01-07

**Authors:** Wladimir Mauhin, Olivier Lidove, Olivier Benveniste

**Affiliations:** 1 Internal Medicine Department, Groupe hospitalier Diaconesses Croix Saint Simon, 125 rue d’Avron, 75020 Paris, France; 2 Sorbonne Université, INSERM, UMR 974, Centre of Research in Myology, Association Institut de Myologie, Pitié-Salpêtrière University Hospital, 75013 Paris, France

**Keywords:** Fabry disease, Antibodies, Inhibition, Phenotype

## Abstract

Fabry disease (OMIM #301500) is an X-linked disorder caused by alpha-galactosidase A deficiency with two major clinical phenotypes: classic and non-classic of different prognosis. From 2001, enzyme replacement therapies with agalsidase alfa and beta have been available. In this letter we underline the different clinical and technical considerations the readers have to be aware of to interpret the results of studies dealing with Fabry disease and anti-agalsidase antibodies. We reaffirm that antibodies preferentially develop in the severe classic Fabry phenotype, which can mislead into interpreting that antibodies are associated with much severe clinical events.

## Dear Editors

We read with interest the letter from Lenders et al. concerning our recent article entitled “Deep characterization of the anti-drug antibodies developed in Fabry disease patients, a prospective analysis from the French multicenter cohort FFABRY” [1, 2]. In the letter, our main message seems to have been eluded: the development of anti-drug antibodies (ADAs) depends on the clinical phenotype (ADA-positivity in classic patients 58.6% vs 6.7% in non-classic patients, *p* < 0.001). Also, with the limits of a time-point study, after stratification on the clinical phenotype, we did not observe any obvious clinical event associated with the presence of ADAs. It is essential to remind that patients with a classical phenotype are more prone to develop a severe renal disease. In our cohort, all the kidney transplanted patients belonged to the classic group, independently from any ADA (see Fig. 1). Also we should have mentioned that 2 ADA-positive patients had benefited from a renal graft before the introduction of enzyme replacement therapy (ERT), suggesting an obvious severe disease prior to the development of antibodies. Concerning the exposure to ERT, the letter mentions that “more ADA-positive patients were treated with agalsidase beta”, which is wrong: as mentioned in our article, there was no difference in terms of seroprevalence in the different treatment group (alfa 30.8%, beta 44.4%, alfa and beta 42.9%, *p* = 0.7). There was also no difference in the mean infused dose received by patients during their whole exposure to ERT (ADA-positive vs ADA-negative patients 0.43 mg/kg vs 0.64 mg/kg, p = ns).

We agree with Lenders and colleagues that purifying IgG subclasses could bring essential information concerning immunogenicity as a first approach. Also, it appears that this has not been performed in the referenced paper [3] where authors used purified total IgGs. We also agree with the authors that ADAs do not possess a mandatory neutralizing activity. This is the reason why we think that inhibition assays should only be performed after a first step using an immune-based assay such as an ELISA. Our goal was to study all ADAs, neutralizing and non-neutralizing. We may have to clarify that we did perform inhibition assay in all the men, contrary to what is mentioned in the letter. As expected, any of the antibody-negative serum was associated with enzymatic inhibition (Fig. 4a). It should also be reminded to readers that there is no consensus for inhibition assay and that the percentage of enzyme inhibition depends on the concentrations of ERT used in the protocol of the inhibition assay. Therefore, there is a need to standardize the protocol and the threshold retained to define inhibition.

In summary, we cannot conclude that antibodies (not only neutralizing) are associated with clinical events in our cohort in this time-point study.

## Abbreviations

ADA: Anti-drug antibodies
ERT: Enzyme replacement therapy
